# Methodological Challenges in Investigating Supracondylar Fractures of the Humerus From a Child’s Viewpoint: Evolution of Study Protocol

**DOI:** 10.2196/21816

**Published:** 2020-11-02

**Authors:** Brittany Tara Lim, Harpreet Chhina, Ian Pike, Mariana Brussoni, Anthony Cooper

**Affiliations:** 1 Department of Orthopaedics British Columbia Children's Hospital Vancouver, BC Canada; 2 Department of Experimental Medicine University of British Columbia Vancouver, BC Canada; 3 Department of Pediatrics University of British Columbia Vancouver, BC Canada; 4 British Columbia Injury Research and Prevention Unit Vancouver, BC Canada; 5 British Columbia Children's Hospital Research Institute Vancouver, BC Canada; 6 School of Population and Public Health University of British Columbia Vancouver, BC Canada; 7 Department of Orthopaedics University of British Columbia Vancouver, BC Canada

**Keywords:** supracondylar fracture, pediatrics, trauma, protocol, injury prevention, child's viewpoint

## Abstract

**Background:**

Outdoor play and risk-taking behaviors, including play at heights, are important to children’s physical, social, and cognitive development. These aspects of play are important to consider when informing prevention policies for serious injuries that commonly occur on play structures. Supracondylar fractures of the humerus (SCH) are the most common type of elbow fractures that result from falls on an outstretched hand among healthy children. Despite being one of the leading causes of admission to the hospital and surgical intervention, the details surrounding the cause of these injuries are often not recorded. Previous research has correlated decreased overall playground safety with higher rates of SCH fractures. Play structure height and the type of undersurface have been identified as potential risk factors for severe injuries, including SCH fractures, in part due to low compliance with safety standards. This paper explores the challenges we encountered designing the study and the resulting insights and methodological modifications we made.

**Objective:**

The aim of this paper is to discuss the challenges related specifically to clinical research in pediatrics and strategies developed to conduct a study that prioritizes the engagement and perspective of children and their families.

**Methods:**

To explore the link between the severity of SCH fractures and children’s behavioral, environmental, and mechanistic factors, we conducted a mixed-methods study.

**Results:**

During phase 1 (the original methodology) from April 2017 to July 2018, there were 58 eligible study participants and 17 were recruited. For phase 2 (the revised methodology) between October 2018 and October 2019, there were 116 eligible participants and 47 were recruited.

**Conclusions:**

The changes in methodology made following the first phase of data collection were effective in our ability to recruit participants. By identifying and addressing challenges pertaining to recruitment and resource limitations, we were able to collect data in a concise manner while not compromising the quality of the data and make for an easily adoptable methodology for other sites interested in participating in the study. We hope that future studies that plan to employ a similar methodology can gain insight through the methodological challenges we have encountered and the way we adapted the methodology to build a more pragmatic approach.

**International Registered Report Identifier (IRRID):**

DERR1-10.2196/21816

## Introduction

Supracondylar fractures of the humerus (SCH) are the most common type of elbow fractures that result from falls on an outstretched hand among healthy children [1,2]. In Canada, the incidence varies by province but has increased over time, accounting for 75% of total pediatric elbow injuries, with a peak incidence among children aged 5 to 8 years [3,4]. SCH vary from simple fractures that heal well with good outcomes by being treated with a cast to those that result in significant disability due to irreparable damage to the neurovascular structures in the forearm. With increased severity of injury, treatments may include surgery, and the potential for complications increases. Early surgical intervention is important in displaced fractures [5,6]. Complications that result from injury include infection, nerve injury, and vascular compromise, which can result in the devastating complication of the Volkman ischemic contracture, leading to lifelong disability [7]. Despite being one of the leading causes of admission to the hospital and surgical intervention [8-11], details surrounding the cause of these injuries are often not recorded. Furthermore, existing literature has not detailed the mechanical and behavioral causes and circumstances leading to injury [12].

Previous research has correlated decreased overall playground safety with higher rates of SCH fractures [13]. Play structure height and the type of undersurface have been identified as potential risk factors for severe injuries, including SCH fractures, in part due to low compliance with safety standards [12,14]. A study comparing falls from playground equipment versus standing height found that falls from playground equipment represented 85% of major fractures [15]. However, these studies did not specifically address SCH fractures but rather a wide range of upper limb fracture types.

Outdoor play and risk-taking behaviors, including play at heights, are important to children’s physical, social, and cognitive development [16-20]. These aspects of play are important to consider when informing prevention policies for serious injuries that commonly occur on play structures. The majority of injuries that result in an SCH fracture in children are thought to occur on playground structures; however, there is limited data supporting this assumption [11,21]. Although falls from monkey bars have been reported as the cause of over 60% of injuries resulting in an SCH fracture, there are limited Canadian data to support this [10,15,22]. It is important to note that despite playgrounds being a common location for injuries among young children, the frequency and severity are relatively low [23]. To inform evidence-based injury prevention policies that take into consideration the aspects of play most important to a child’s development, more research on SCH fractures is needed to gain a better understanding of the specific mechanisms and child-related factors surrounding injuries.

To explore the link between the severity of SCH fractures and children’s behavioral, environmental, and mechanistic factors, we conducted a mixed-method study among children presenting to the Department of Orthopaedics at the senior author’s center from June 2017 to the present. We used qualitative interviews with children combined with the use of visual aids, such as photographs, and quantitative analysis of playground structures comparing them to the safety standards [24-27]. This combination of methods has been shown to assist children who have experienced elbow fractures in sharing their viewpoints in a clinical setting [28].

An important and increasingly relevant perspective in clinical research is that of the child’s viewpoint. For instance, efforts to understand a traumatic event from a child’s perspective are important in clinical research so as to capture relevant aspects of the events leading up to the injury that may be overlooked or missed in the relay of the incident by parents and caregivers [29,30]. Important ethical considerations need to be made to the methodology when conducting patient-oriented research, particularly with children. For example, the appropriate age at which a child can give consent or refuse assent must be considered in the recruitment process. Additionally, we need to be careful around questions that may elicit negative emotions in children [30]. Through purposeful integration of children’s perspectives and child-friendly methodologies into research, relevant factors pertaining to the injury may be identified that are often overshadowed in routine, adult-focused elicitation [25,28,31].

This paper explores the challenges we encountered and the resulting insights and methodological modifications we made. We will discuss the challenges related specifically to clinical research in pediatrics and the strategies developed to conduct a study that prioritizes the engagement and perspective of children and their families. The aim of this manuscript is to describe the progression of the mixed-methods study protocol from a detailed qualitative approach to a more pragmatic approach.

## Methods

### Phase 1. Original Methodology

Data collection for the study was designed to coincide with children’s routine 3-week, 6-week, and 12-week postinjury appointments at the orthopedic clinic at British Columbia Children’s Hospital. Once the study received ethics approval (#H17-00561), eligible study participants were identified by the senior author. A designated research assistant assented child participants while consent was obtained from the parents/legal guardians at the first visit to the orthopedic clinic. Consented participants were given a GPS camera and a prepaid mailing envelope for the return of the camera. They were instructed to take photographs of the play structure where the injury occurred. This required the families to go back to the site of the injury to take the photographs. They were provided with training on basic photography skills and made aware of privacy concerns during photography. At this point demographic and basic injury data were collected by asking the participants’ parents in an informal interview setting. Fracture classification and treatment plan were collected through a medical chart review.

At the 6-week follow-up, a photo elicitation interview (PEI) was conducted using the participant-generated photographs to discuss the mechanism of injury, site of injury, and injury experience. The children were encouraged to describe how they fell and other details around their injury and recovery, aided by the photographs they took. Interviews were audio recorded and transcribed verbatim using a professional transcription service. The transcripts were reviewed using a framework analysis that involved interpretation, thematic identification, charting, and consensus codes from two reviewers [32,33]. Treatment updates and complications were also recorded from medical chart review after every clinical follow-up. At the 12-week follow-up, participant medical charts were used to log final injury-related outcomes and complications. No participant interaction was necessary.

Using the GPS cameras, the exact location of the injury was extracted from geo-tagged photographs taken by the injured children. A research assistant identified the exact playground equipment where the injury took place using the images taken by the participants combined with the information collected in the interview. This research assistant visited each of these injury sites and took measurements of the play structures involved in the injury. Several measurements were taken including the surface depth of the terrain, height of the play structure platforms, and handlebars. Measurements were taken using basic tools including a ruler, tape measure, and soil probe. The results were compared with the standards provided by the Canadian Standards Association, which provides detailed information about materials, installation, and strength of the equipment; surfacing, inspection, maintenance, performance requirements, and access to the playground; play space layout; and specifications for each type of equipment [34].

### Identified Challenges

#### Participant Recruitment

The main concerns voiced by participants and families that declined participation were related to the time investment required by the families. In particular, returning to the site of the injury to take photographs and partaking in the PEI were identified as demanding. To improve participation, changes were made to the methodology based on these concerns.

#### Resource Limitations

Constraints in resources were also barriers in following the methodology of this study. This methodology required purchasing GPS cameras to take photos of the playground sites. Additionally, special training was required for staff to conduct the PEIs. Likewise, analyzing the qualitative data was time intensive. The interviews were scheduled to take place at the time of the participant’s regular clinic follow-ups to save families from an additional visit, but a trained interviewer was not always available at that time. In those cases, participants and their families were asked to return to the hospital to complete the interview.

### Phase 2. Revised Methodology

**Figure 1:**
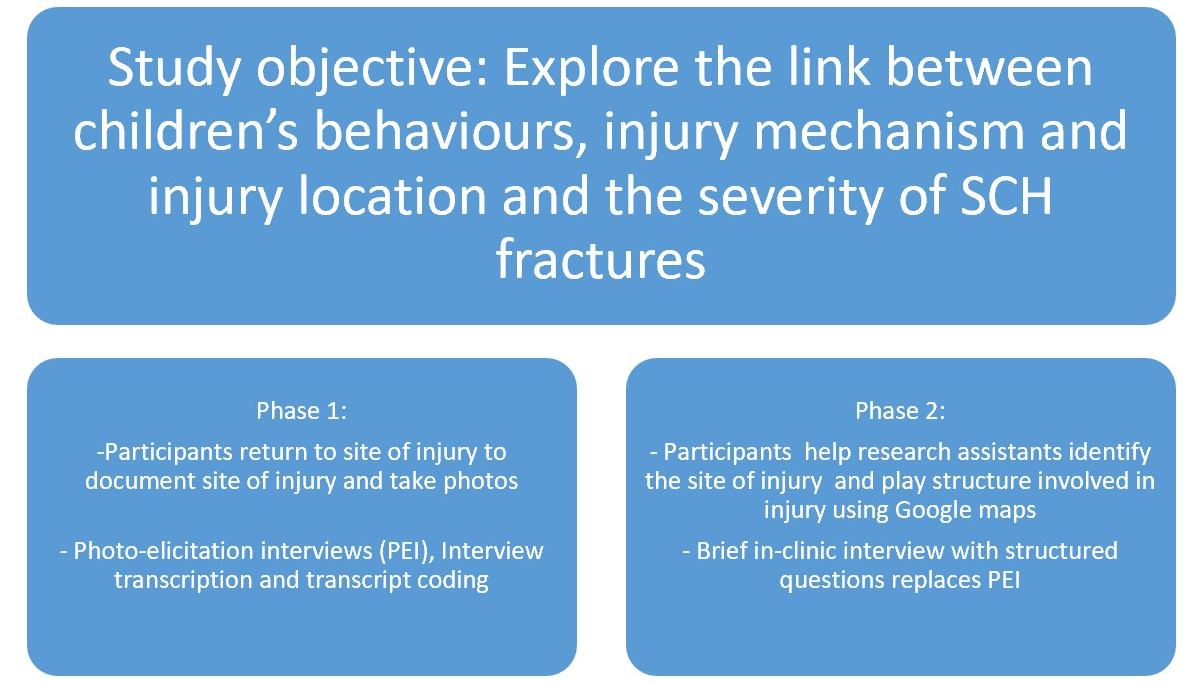
Study objective with the key components from each phase of the study.

The study methodology was revised to overcome challenges identified during phase 1 while addressing the same research questions. Figure 1 demonstrates the relationship between the two phases of the study. A summary of modifications can be seen in Table 1 and Multimedia Appendix 1. At the first visit to the orthopedic clinic, children who consented to participate were asked a series of questions pertaining to their injury. Data collection except for the measurement of the play structure was completed at the time of recruitment. The participant was asked to describe the location of the injury event and the play structure involved. The family provided the address, at which point the research assistant typed that into Google Maps. Using satellite view, the participant indicated the exact play structure where the injury occurred. The research assistant then took a screenshot image of the structure and the street view of the location to make the identification of the play structure easy when going to the site to complete the measurements. This ultimately allowed for the elimination of GPS cameras and the need for the participant to return to the site of injury. This change in methodology increased efficiency, reduced resource requirements, and maintained feasibility for both the research study and the participants and families. Families were not pressured to return to the site of injury between clinic follow-up appointments to take photos of the site. This action reduced the financial requirements for having expensive cameras.

If the site of injury or play structure was not visible using Google Maps, the participant and their parent or legal guardian were asked to return to the site and take photographs using their personal photo-taking device in order to identify the exact play structure for measurement. The participants were informed of privacy concerns during photography, and instructions on what to capture in the photos were described. Participants were then asked to describe the mechanism of injury through a short in-clinic interview transcribed by the research staff. At the first visit, demographic data, fracture classification, treatment plan, and basic injury data were also collected. At the 6-week or 12-week clinic follow-up, recruited participants who were injured on a play structure not visible using Google Maps were asked to send photographs of the play structure involved in the injury via email.

In phase 1, specific training was required for the research assistant to be able to conduct and review the PEI. Phase 2 alleviated the need for extensive preparation and training to conduct the interviews because of the more structured setting. The extensive amount of time required to transcribe, code, and analyze the qualitative data was eliminated.

Given that the aim of the interviews remained the same in both phases of the methodology and the questions were focused on uncovering specific aspects of the child’s memory of the injury, the research assistant asked questions and provided prompts as necessary to help the participant with elicitation of the injury. Based on the preliminary analysis of the data collected in phase 1, we were able to select the questions in the PEI based on the questions that elicited the most complete responses during the PEI. Multimedia Appendix 1 includes more details on the changes made to the questions through the evolution of the protocol.

Some children had difficulty articulating the events that lead to the injury or simply said they did not remember. To aid the elicitation of the injury events in these cases, the research staff demonstrated different arm motions and asked participants to identify which motion was most like their experience. Research staff were intentional in clarifying these memories while remaining impartial to avoid influencing the responses.

**Table 1:** Overview of modifications to the methodology.

Phase 1: Identified challenges	Phase 2: Modifications to the methodology
Recruitment	Condensing data collection into one clinic visit to reduce the need for research follow-up at every clinic visit
Time investment	Use of Google Maps to identify play structure where injury occurred to eliminated need for participants and their families to return to the site of injury
Resource limitations	Replacing the detailed PEI^a^ with a short in-clinic interview with structured questions and therefore no longer needing GPS cameras

^a^PEI: photo elicitation interview.

## Results

During phase 1 (the original methodology) from April 2017 to July 2018, there were 58 eligible study participants and 17 were recruited. For phase 2 (the revised methodology) between October 2018 and October 2019, there were 116 eligible participants and 47 were recruited. The results from this study will be published in a separate manuscript.

## Discussion

### Summary

The progression of the study methodology has been integral to advancing this research because patient engagement and input is valuable in clinical research settings [29]. The identification of challenges such as recruitment, time investment, and resource limitation enabled us to see the need to modify the methodology. Through identifying the most relevant aspects of data collection and modifying methodology to collect these data, we have taken a more pragmatic approach and shifted some data collection methods from qualitative to quantitative.

In the first phase of data collection, qualitative data pertaining to the injury experience from the child’s perspective were prioritized. However, as phase 1 was completed, it became apparent that amendments to this methodology could reduce resource investment in data collection and analysis while still answering the research question. In doing so, we have lost some of the qualitative data that were collected through the photo elicitation interviews. This could represent a potential limitation to the modified research methodology as we were not be able to go as in depth on the behavioral, emotional, and mechanistic factors influencing the events surrounding the injury from the child’s perspective.

Challenges in clinical research can provide opportunities to explore new methodological approaches for data collection, recruitment, and participant interaction. Steps toward addressing resource investment and increasing patient participation and hence the recruitment rate were undertaken in this study. Through the pediatric elbow fracture study, we have shown the feasibility of making modifications to methodology to an ongoing study to reduce patient and resource burden. Our modification of methodology did not compromise the quality of our data.

### Recruitment

The increase in recruitment rate from 0.29 to 0.41 is an indication that the modifications to the methodology were effective in addressing the concerns of eligible participants and their families.

To address the concerns voiced by participants and their families that participated and declined participation, a more pragmatic methodology was devised that allowed for data collection while reducing demand on the participating families. Consideration of the time commitment that the study demanded of participants and their families was important, as it led to our understanding that this requirement of the study impacted recruitment.

### Google Maps

The use of Google Maps satellite view enabled the research assistant to identify the exact location of the injury event with the participant and the family at their clinic visit. However, a limitation to the use of Google Maps was if the play structure was not visible using street view, then the participant would need to return to the site to take pictures of the exact structure where the injury occurred. In these cases, we asked the participants’ families to take photos on their personal devices and send the photos to the research team by email.

### Time Investment

Condensing data collection into one clinic follow-up appointment was an effective change to the methodology because this enabled the recruitment of eligible participants at any of their clinical appointments and alleviated some of the time investment that the original methodology required.

### Resource Limitation

The PEIs used photographs taken by participants to help overcome age-related linguistic and cognitive barriers for young participants [31]. However, it required participants and their families to return to the site of injury to take photos in addition to the time commitment of the interview itself. This was time consuming for participants and their families, and it was felt that the necessary information could be collected through a shorter and more structured interview as opposed to a semistructured interview. There were concerns that the change in methodology would affect the quality of data. However, the data from the original and revised methodology were found to be comparable.

Therefore, we began performing brief in-clinic interviews that captured the child’s account of events leading to injury. Using focused questions informed by our phase 1 interviews, we were able to obtain detailed accounts of the injury from the child’s perspective. The questions were centralized around the mechanism of the injury and a description of the play structure or location of where the injury took place. From this, we were able to modify our questions that specifically addressed our research inquiry without having to do a more detailed interview. By replacing the PEI with a more condensed and structured set of questions that could be answered during the participants’ regular clinic follow-ups, we were able to collect important information on the mechanism of injury from a child’s perspective while alleviating resource limitations associated with transcribing and coding the interview and training for staff to conduct the interview.

In clinical research, it is important to address barriers affecting recruitment and patient involvement and formulate pragmatic solutions to retain participation. By accommodating the needs of participants and their families, the recruitment rate was improved for the ongoing elbow fracture study. This experience has emphasized the importance of taking into consideration what participants and their families value most when being involved in research studies. Participant and family participation and input in the development of guidelines and methodology can provide insight to clinicians that could be otherwise overlooked [35]. This insight is valuable during the design phase but is still important to consider at all stages of the study, as demonstrated in the methodology outlined in this manuscript.

More Canadian research is needed to identify and evaluate the safety of playground structures including specific mechanisms and child-related factors surrounding elbow fractures to inform prevention policy. Addressing resource constraints was important to ensure that the methodology was not only feasible and sustainable for our site but also to facilitate this as a multicenter study resulting in a larger sample size.

### Conclusion

The changes in methodology made following the first phase of data collection enhanced our ability to recruit participants, collect data in a concise manner while not compromising the quality of the data, and design an easily adoptable methodology for other sites interested in participating in the study. We hope that future studies that plan to employ a similar methodology can gain insight through the methodological challenges we have encountered and the way we adapted the methodology to build a more pragmatic approach.

## Appendix

Multimedia Appendix 1 Iterations of the questions asked in clinic.

## Abbreviations

SCH: supracondylar fractures of the humerus
PEI: photo elicitation interview
