# Supplementary material for: Methodological Challenges in Investigating Supracondylar Fractures of the Humerus From a Child’s Viewpoint: Evolution of Study Protocol
Source: JMIR Res Protoc. 2020 Nov 2;9(11):e21816. doi: 10.2196/21816 (PMC7669438; doi:10.2196/21816)
Supplement: Multimedia Appendix 1 [file resprot_v9i11e21816_app1.docx]

Appendix

Phase one:

For the first phase of the methodology semi structured questions were used in a formal interview setting outside of their clinical visit. Research staff was provided with the questions listed below. The list of questions was provided in the event that the participant required a prompt in elicitation of the injury.

**Parent introduction:** Our interview today will take approximately 30-45 minutes.

As stated in your consent form, each interview will be audio recorded and you and your child may stop at any time.

We prefer to interview your child alone to ensure they are participating independently, but you may be in the room if you or he/she wishes. Again, all parts of this study are voluntary and you or your child can choose not to answer any question or stop the interview at any time.

All information will be kept confidential and no identifying information will be included in any results.

This study will have no bearing on your child’s current or future care.

**Participant introduction:** Hi [participant’s name]. I am going to ask you to tell me about how you broke your arm using the pictures you took. I have printed your pictures so we can look at them together. I will also be asking you some questions, is this okay with you? Everything you tell me is confidential, that means that no one will know about it except you and me and my team. You can stop at any time and no one will be mad at you. You also don’t have to answer any questions you don’t want to.

The following are prompting questions used to help facilitate the elicitation of the injury in the event that more information on the mechanism of injury is needed:

**Can you tell me something about yourself?**

Icebreaker: snack, age, school, favourite subject, siblings, cast, etc

Do you want to see your pictures?

1. Lets look at the first picture.
2. Can you tell me about this picture
3. Why did you take this picture?
4. Why did you think that this picture is important in relation to your fracture?
5. Can you tell me about how you broke your arm?
6. Where were you when you broke it?
7. What time was it?
8. What did you hear?
9. What were you doing when you broke it?
10. Do you do this a lot?
11. How often?
12. Who were you with?
13. Was anybody else around?
14. How did you fall?
15. Can you show me?
16. Why do you think your injury happened?
17. Do you think anything could have been done to stop it from happening?
18. What happened after you fell? Did you go to the hospital right away?
19. How long were you in the hospital?
20. Do you still do that activity?
21. Are you scared or nervous to do that activity again?
22. What will you do next time so you don't break your arm again?
23. Do any of your photos describe how you felt when you broke your arm?
24. Or how you felt after?

Phase two:

In phase two of the methodology, participants were approached at their scheduled clinic visit. Research staff asked each participant to answer the following questions (with the assistance of their parents if necessary):

- Are you right or left hand dominant? Which hand do you use to write with?
- Where did the injury occur?
  - GPS location
  - General description
- What were you doing when you fell?
- How did you fall?
  - Can you show me with your arm how you landed on the ground?
- If injury occurred on a play structure: can you describe the play structure you were on?
  - Colour
  - Type of play structure
  - Relative location to other play structures
